# Supplementary material for: Are carbon emissions trading and green financial instruments synergistic? -Comprehensive quantitative research based on content analysis
Source: PLoS One. 2024 Mar 7;19(3):e0298601. doi: 10.1371/journal.pone.0298601 (PMC10919601; doi:10.1371/journal.pone.0298601)
Supplement: S1 Data — (ZIP) [file pone.0298601.s001.zip › Data/地级市Z_F工作报告.zip!/╜¡╬≈/╕o╓▌╩╨/└┤╫╘╣1⁄2╓┌║┼í╛┬φ┐╦╩2╛▌═°í┐_2015.docx]

各位代表：

现在，我代表市人民政府向大会报告工作，请予审议，并请各位市政协委员和列席会议的同志提出意见。

一、2014年工作回顾

2014年是我市全面深化改革的开局之年，也是实施“三大战役”、建设幸福抚州的提升之年。一年来，我们主动适应新常态，保持战略定力，狠抓发展升级，实现了持续稳定健康发展。全年完成生产总值1036.77亿元，增长9.8%，首跨千亿元大关。财政总收入150.07亿元，增长14.9%，一般公共预算收入116.38亿元，增长15.8%。规模以上工业增加值315.2亿元，增长11.7%。固定资产投资957.25亿元，增长20.5%。社会消费品零售总额379.5亿元，增长11.7%。实际利用外资3.3亿美元，增长13.4%，外贸出口15.39亿美元，增长23.5%。城镇居民人均可支配收入23101元，增长9.6%，农村居民人均可支配收入首次突破万元，达到10410元，增长11%。

――“三大战役”持续升温，发展升级态势更为强劲。工业经济加速崛起。工业实现税收41.8亿元、增长28.9%，高於财政收入增幅14个百分点，工业利税增幅全省第一。新引进5000万元以上工业项目290个，实际进资344亿元，其中亿元以上项目152个，实际进资273亿元。新增规模以上工业企业140户，总数达到900户。完成工业固定资产投资560亿元，增长12.7%，全市12个工业园区实际开发面积达到49.94平方公里。县域经济快速发展。县县财政收入过6亿元，南丰、黎川、崇仁和抚州高新区财政收入首次突破10亿元，东乡县财政收入首次突破20亿元。抚州高新区汽车及零部件、崇仁变电设备和金溪香料获批省重点产业集群，临川抚北工业园区获批省级有色金属加工产业基地，广昌、宜黄工业园区汽配产业加速聚集。南城、崇仁工业园区扩区调区获省政府批复。乐安工业园区列入省级工业园区管理。资溪生态游、南丰桔园游受到市场热捧。跑项争资取得重大突破。杭南长客专抚州段建成通车，向莆铁路货运快速班列试运行。新开建东乡―昌傅、广昌―吉安、船顶隘―广昌三条高速，抚州―金溪、资溪花山界―里木、南昌―宁都高速乐安段进展顺利。抚州电厂建设快速推进，抚州机场筹建前期工作正式启动。原中央苏区县全部执行西部地区中央预算内投资政策，一大批重大项目和事项列入《赣≥粤原中央苏区振兴发展规划》。特别是省委、省政府《关於支持抚州深化区域合作加快发展的若干意见》正式出台，这是首个专门为抚州量身打造的支持性文件，把“融入南昌、对接海西，建设向莆经济带”上升为省级战略，具有里程碑式的意义。

──转型升级步伐加快，经济运行质量进一步提升。产业和收入结构进一步优化。服务业占GDP比重增加1个百分点，对经济增长贡献率提高2.92个百分点。税收收入增幅全省第三，占财政总收入比重达83.2%，提高1.4个百分点，高出全省平均水平1.9个百分点，排名再进一位，列全省第五，万元GDP综合能耗下降3.5%，六大高耗能行业综合能耗下降8%。全市工业园区亩均投入、产出提高21.6%和14.7%。产业竞争力进一步增强。农业稳产提质，粮食总产量289.24万吨，实现“十一连丰”，新增省级农业产业化龙头企业8家。旅游强市战略深入推进，全年接待国内外旅游人数1650万人次，旅游收入133亿元，分别增长35%和38%，梦湖景区被评为国家4A级景区。电子商务蓬勃兴起，交易额106.7亿元，增长226.5%，盈川实业成为全省首家在上海股交中心挂牌上市的电商企业，黎川县东鑫电子商务园被授予全省第二批电子商务示范基地，抚州高新区引进电商企业40家。建筑业加速振兴，实现产值260亿元，增长21%，新增7家建筑企业晋升一级资质。金融业加速发展，人民币各项存款余额增幅列全省第二，新增贷款超过150亿元，增幅列全省第一，“财园信贷通”贷款21.9亿元，率先在全省建立中小企业还贷周转金制度，赣州银行、南昌银行、兴业银行在我市设立分行或支行，九江银行、上饶银行、浦发村镇银行、南昌农商银行在县(区)新设分支机构或村镇银行，金溪农商行顺利开业，红塔证券、长江证券在我市设立营业部，苍源种植有限公司在“新三板”挂牌上市，抚州海峡钢铁交易所获批筹建，政策性农业保险效益提高，商业保险深度参与城镇居民大病补充医疗保险。创新能力进一步提升。新组建4个省级工程技术研究中心，新培育认定10个市级工程技术研究中心和15家高新技术企业。4项优秀科技成果获省科学技术奖，为历年获奖最好成绩。抚州市青年(大学生)创业孵化基地获团中央命名首批“全国青年创业示范园区”。金溪香料协同创新体列为省重点扶持培育的13个协同创新体之一。市政府与中关村管委会、南昌大学、江西中医药大学、东华理工大学签订合作协议，抚州高新区与南昌大学签署共建抚州研究院合作协议，4家农业产业化企业与中山大学达成初步合作协议。高新技术产业增加值60.28亿元，增长17.1%。

──全面深化改革蹄疾步稳，发展活力明显增强。深化行政审批制度改革，接国务院、省政府取消和下放的行政审批209项，取消20项，下放121项，精简率68%。开展行政权力梳理，对保留的231项进行了流程再造。全面实行注册资本认缴登记制和简化经营场所登记条件改革，新登记注册企业5087户、增长19.53%，新增注册资本187.98亿元、增长50.39%。全面推动农村改革，扎实推进农村土地确权登记工作。深化教育领域综合改革，获批建设全国中小学教育质量综合评价改革实验区和抚州基础教育综合改革试验区。大力推进南城县省直管县改革试点。

──城乡建设扎实推进，人居环境显著改善。深入开展“四城同创”，已成功创建“国家园林城市”、“国家森林城市”和“省级卫生城市”。在市中心城区实施91个重点项目，完成投资24亿元，其中老城区改造投入近7亿元。启动城市棚户区改造8431户、面积101.82万平方米,已签订安置协议7123户，开建安置房6713套，货币安置1718户。投入近5亿元，完成685个新农村建设村点的村庄整治任务。启动实施37个乡(镇)的镇村联动建设，辐射带动465个周边村庄。关停36家非法电镀、携工、小造纸等企业，开展了农村环境卫生综合治理、水库水质污染和禽畜养殖污染专项整治行动。投入20.8亿元，实施310个农田水利建设项目。投入2.8亿元，完成农村公路建设里程782公里。全市城镇化率提高1.4个百分点，达到43.5%。

――民生保障力度加大，社会大局和谐稳定。全面完成省政府下达的50件民生实事。新增城镇就业4.58万人，转移农村劳动力6.59万人。推进城乡居民养老保险制度并轨，开展最低生活保障资金城乡统筹试点，启动了全国养老服务业综合改革试点。新建公租房开工6970套，基本建成1669套，新建廉租房开工2224套，基本建成618套。启动国有林场危旧房改造968户、垦区危房改造1965户和国有工矿棚户区改造584户。高考成绩再创辉煌，首次包揽全省文理科第一、二名，教育资源进一步扩大，增加中小学学位3.2万个。卫生、文化新闻出版、广播电影电视、人口和计划生育、体育、质量技术监督、食品药品监督、国资监管、工商行政管理、民兵预备役、国防动员、人民防空、民族宗教、扶贫移民、外事侨务、涉台事务、审计、统计调查、防震减灾、气象、水文、档案、地方志、妇女儿童、老龄、残联、红十字会、驻外办事处等工作有了新的进步。进京、赴盛来市信访量实现“三个大幅下降”，社会治安和安全生产形势进一步好转。

――作风建设长抓不懈，政府自身建设不断加强。扎实开展党的群众路线教育实践活动，针对“四风”突出问题，落实了整改措施，完善了长效机制。深入推进党风廉政建设，严肃查处了一批违纪违法案件。认真办理178件市人大代表建议和302件市政协提案，满意率99.5%。对整顿机关作风、整治施工环境、重大招商引资和重点项目落实情况，进行了专项效能监察，查处和曝光一批损害发展环境的典型案件。

各位代表，回顾过去一年，我们砥砺奋进，取得这些成绩，殊为不易。这是省委、省政府和市委科学决策、坚强领导的结果，是市人大、市政协有效监督、大力支持的结果，是全市上下团结一心、合力攻坚的结果。在此，我谨代表市人民政府，向全市广大干部群众，向各民主党派、工商联、无党派、新阶层、人民团体、社会各界人士，向中央、省驻抚单位，驻抚部队和武警、消防官兵，向外来投资者和建设者，向所有关心、支持和帮助抚州发展的同志们、朋友们，表示衷心的感谢和崇高的敬意!

在看到成绩的同时，我们也清醒地认识到存在的不足和问题，主要有：发展不足的矛盾仍然突出，经济总量孝人均水平低的状况尚未得到根本改变，发展面临的要素制约和资源环境约束凸显，经济下行压力增大，实体经济面临困难较多，产业集聚度不高，产业层次较低，产品附加值不高，企业实力较弱，市场竞争力不强，科技创新能力不足，尤其是高、精、尖人才匮乏，发展环境仍然不够理想，社会矛盾频发多发，社会稳定的基础尚不牢固，“既不出事也不干事”、“为官不为”的思想在部分干部中滋长。对此，我们将高度重视，著力破解。二、2015年工作安排

今年是“十二五”规划收官之年和“十三五”规划的谋篇之年，也是全面深化改革和发展升级的关键之年，全面建设法治国家的开局之年。做好今年各项工作，对於实现建设殷实、文明、和谐的幸福抚州总目标，与全国、全省同步全面建成小康社会的宏伟事业，具有极其重要的意义。

今年政府工作的总体要求是：全面贯彻落实党的十八大、十八届三中、四中全会和中央经济工作会议精神，以邓小平理论、“三个代表”重要思想、科学发展观为指导，深入学习贯彻习近平总书记系列重要讲话精神，紧紧围绕省委十六字方针，坚持稳中求进工作总基调，以深化区域合作为主线，以改革创新为动力，全力打造以区域升级、产业升级、开放升级和创新升级为核心的“三大战役”升级版，著力保持经济持续较快增长良好势头，切实提升经济发展的质量和效益，不断增进民生福祉，促进社会和谐稳定，在新常态下展现新作为，实现新发展。

今年全市经济社会发展的主要预期目标是：生产总值增长9.5%，达到1140亿元，财政总收入增长11%，达到166.6亿元，规模以上工业增加值增长12%，达到353亿元，固定资产投资增长18%，达到1130亿元，社会消费品零售总额增长12%，达到425亿元，出口总额增长8%，达到16.6亿美元，实际利用外资增长15%，达到3.8亿美元，城镇居民人均可支配收入增长10.5%，达到25520元，农村居民人均可支配收入增长11.5%，达到11600元。城镇化率提高1.5个百分点，达到45%。

围绕上述目标，重点抓好八个方面的工作：

(一)全力抢抓深化区域合作新机遇。

紧紧围绕“融入南昌、对接海西、建设向莆经济带”，用活用足用好政策，加快推动区域升级和开放升级。

全力推进重大项目建设。加快推进抚州―金溪、资溪花山界―里木、南昌―宁都高速乐安段、船顶隘―广昌及其西延线广昌―吉安、东乡―昌傅等高速公路建设。尽快启动昌抚城际大道建设项目前期工作，力争项目列入拾十三五”规划。加快江西出入境检验检疫局抚州办事处检测中心项目建设，力争年底前完成主体工程，积极争取设立抚州海关。积极争取国家、省发改委尽快审批廖坊灌区二期可研和工程建设资金报告。积极协调配合、加快推进抚州电厂建设 诛仙小说。抓装十三五”规划编制的机遇，积极包装、储备、申报一批项目，力争鹰梅铁路、吉抚武铁路、抚州机尝抚河通航工程、抚金与昌东高速连接线、崇仁―宜黄―南城高速、抚州电厂二期等重大项目列入国家、省计划。

著力打造产业转移重要承接区。围绕“一核三圈”产业格局，加大≥台招商特别是对台招商力度，力争≥台招商项目个数和实际进资增长20%以上。同时抓爪昆高铁开通机遇，加大江浙沪地区招商力度。突出抓好环境招商，认真执行国务院关於全面清理规范税收等优惠政策的工作部署，著力营造良好市撤境，不断提高办事效率和服务水平。力争全年新引进5000万元以上工业项目260个以上，实际进资380亿元以上。积极做好中央统战部今年在我市举办“光彩事业抚州行”活动的筹备及招商工作，争阮动签约投资额过千亿。

努力建设全省区域合作发展创新区。积极争取省政府尽快批复昌抚合作示范区规划，按照“改革创新先行区、城乡统筹试验区、赣≥合作示范区、向莆经济带重要支点”的战略定位，以临川区云山镇为中心，建设昌抚合作示范区，加快昌抚同城化进程，深化与南昌在各层面的合作，争取实现昌抚金融同城化和电信同城化，加快推进昌抚城区公交客运，加密抚州至昌北机场城际快巴。依托向莆铁路货运班列开行，积极推进与莆田口岸深化合作，推动外贸通关便利化，逐步实现“一次申报、一次查验、一次放行”，切实降低企业物流成本。扩大本地产品出口和生产型企业出口，力争生产企业出口占比达40%以上，实际利用外资现汇占比达30%以上。

大力推进县域经济发展。支持县(区)在更宽领域、更深层次参与区域分工合作，竞合县外资源，助推发展升级。支持县(区)坚定不移地走特色兴县、产业强县道路，因地制宜发展主导产业，坚持不懈做大产业集群。支持推动扩权强县，最大限度地为县域经济发展“松绑给力”，赋予县(区)更多发展自主权，充分调动县(区)加快发展的积极性和主动性，深入推进南城县省直管县改革试点。创新县域发展分类考核评价机制，改进考核方法手段，加强考核结果运用，更好地发挥考核评价“指挥棒”作用，引导鼓励各县 (区)特色发展、绿色发展、创新发展、科学发展。

(二)加快释放全面深化改革新动力。

坚持向改革要红利，以更大的决心和力度突破机制体制束缚，激发发展升级活力。

加快推进简政放权和市朝改革。加快推进政府机构改革和职能转变，科学界定政府和市场的边界，进一步向基层、向市场下放权力，充分激发基层和市场主体的发展活力。重点推行“三单一网”，完善市县“并联审批”工作。认真接落实好国务院、省政府取消和下放的行政审批事项，及时跟进省行政审批制度改革步伐，确保已取消下放的审批事项落到实处。进一步简化登记手续，放宽住所(经营场所)条件，推进以电子营业执照为支撑的全程电子化登记管理方式。积极探索“三证合一”制度，放大工商登记制度改革效应。深化价格体制改革，落实好“减、放、改”工作机制，规范政府定价行为。

加快推进财政投融资体制改革。深化公务消费一体化监管改革，稳步推进部门预算编制、政府债务管理和公车改革。进一步放宽投资准入，推行PPP模式，引导民间资本投资基础设施、基础产业、社会服务等重点领域。加快市城投(集团)公司实体化改革步伐。

加快推进农村改革。完成农村土地承包经营权确权登记颁证工作，加快推进土地承包经营权流转、土地征收和宅基地制度改革试点工作。加快建立覆盖县、乡、村的农村土地承包经营权交易平台和网络，促进土地向新型经营主体集中。积极做好农村集体产权股份合作制改革试点工作，保障农民集体资产分配权。切实抓好林权制度配套改革，进一步完善国有林场经营机制。

加快推进国资国企改革。进一步完善现代企业制度，规范国企管理人员薪酬待遇。大力发展混合所有制经济，鼓励支持国有资本、集体资本、非公有资本、域外资本等交叉持股、融合发展。加快国有企业股份制和集团化改造，鼓励赣东路桥集团公司、建筑设计院、规划设计院、水电勘测设计院等单位改制，推进保安公司政企、政资分离。

在抓好上述改革的同时，按照上级总体部署，统筹推进其他各项改革。

(三)坚定迈出产业转型升级新步伐。

坚持扩量与提质、特色与创新并举，著力打造现代产业体系，提高产业整体竞争力。

――加快新型工业化进程。突出抓好“四个发展”：

集聚发展。围绕打造一批在全省有规模、有特色、有比较优势和较强竞争力的产业集群，狠抓“三提”：项目建设提速，加快推进一批签约项目、开工项目、在建项目和增资扩产项目，力争工业固定资产投资增长30%以上。产业招商提质，突出主导产业和特色产业集群招商，力争新引进项目中，主导产业项目占比达到85%以上。企业帮扶提效，进一步扩大还贷周转金规模，加大信贷投放力度，力争年内新增存贷款双双过200亿元。加大扶优扶强和扶持小微企业力度，推动小微企业升规、规上企业做强做大。力争新增规模以上企业100户、总数突破1000户，年主营业务收入过20亿元的企业达到20户以上。

集约发展。集约节约利用工业园区土地，统筹安排各类建设用地，科学制定入园项目投资强度、投资进度等刚性指标。进一步完善集约节约用地的激励和约束机制，鼓励建设多层标准厂房，鼓励企业技改扩大产能，依法依规清理闲置低效工业用地。力争工业园区单位面积投资和产出强度分别提高18和12个百分点以上，著力提高企业工艺技术水平和装备水平。

创新发展。积极探索和推进科技协同创新模式，加强政府、企业与高校及科研院所的战略合作。加大高新技术企业培育力度，力争新增高新技术企业10户以上。开展百企创新暨转型升级试点，著力培育一批龙头骨干科技创新示范企业和科技型小微企业。支持创建高新技术产业化基地和工程技术研究中心，力争抚州高新区成功升级国家级高新区。加大新能源汽车推广应用力度。建立健全多元化科技投入体系，确保市、县(区)财政科技拨款占地方财政支出比例不低於2%，力争全社会研发投入占生产总值比例达到0.8%以上。力争高新技术产业增加值增长25%以上。

融合发展。积极推进工业化和信息化“两化”深度融合，深入实施“个十百千万”工程，积极培育“两化”深度融合示范企业，组织开展企业信息化推进试点，抓好新一代物联网技术在重点企业的广泛应用。推进产城融合，加大“园区办社会”力度，力争全市工业园区就业和居住人口分别增长8%和12%。调整控制性详细规划及有关专项规划，变功能单一的工业“区”为功能复合的产业“城”。支持抚州高新区打造全市产城融合示范区。

――大力发展现代农业。突出抓好“六化”+“接二连三”：

“六化”：产业规模化，推进适度规模产业化经营，变生产“碎片化”为“规模化”、“提篮小卖”为“车拉船载”。产品特色化，做强五大“中国之乡”特色产业，做大新型特色产业。生产智能化，运用机械化和信息化技术，装备、改造和提升传统特色农业。营销品牌化，大力实施品牌带动战略，实现“品牌营销、优质优价”。技术标准化，以无公害、绿色和有机食品标准化生产示范基地为牵引，尽快制定一系列技术标准体系。组织现代化，采劝龙头企业+专业合作社+农民”等多种形式，促进产加销有机融合和全程组织现代化。

“接二连三”：大力发展以农产品加工业为主的第二产业、以休闲观光农业为主的第三产业，延长农业产业链，提高农业的比较效益。一方面，依托我市农业基础，重点发展食品工业、生物医药和农产品精深加工业，切实提高农产品加工转化率、资源综合利用率和市场占有率。大力实施食品产业强市规划，努力把我市的绿色食品产业打造成千亿产业。著力培育龙头企业，积极引进大型农业企业集团，带动我市农产品加工业跨越式发展。另一方面，充分发挥现代农业资源和生态环境优势，依托区域文化、民俗文化、宗教文化和历史文化，结合森林城乡创建和城乡居民休闲需求，大力发展休闲观光农业。

按照“六化”+“接二连三”的发展路径，大力推进与≥台地区现代农业和食品产业的交流合作，不断深化政府与院校、科研单位在发展现代农业方面的合作，支持推动校企、院企合作。抓好11个县(区)现代农业示范园区建设，力争当年入园企业新增投资额超过12亿元，入园项目实现产值20亿元。

――大力发展现代服务业。突出做好“提升”和“培育”两篇文章：

提升发展传统服务业。提升发展旅游业，推进临川文化旅游产业集群和资溪旅游产业集群建设，鼓励各地旅游产业集群式发展，支持资溪大觉山创建国家5A级旅游景区，其他县(区)创建国家4A级旅游景区，充分利用微信等新兴媒体开展系列旅游宣传活动，打响“华夏梦都、才子之乡”宣传品牌。提升发展物流业，加快建设抚北综合物流园，推进东乡渊山岗物流园、南城海西物流园、黎川赣≥物流园、广昌物流仓储配送中心等物流产业集聚区，以及抚州国家粮食储备库扩建等仓储物流设施建设，引进培育大型物流集团及第三方、第四方物流，支持正广通供应链管理有限公司在抚州打造物流集团总部，鼓励大型物流企业和物流园区建设物流信息平台。提升发展商贸业，加快重点商品交易市场建设，推动现有大型交易市场升级，支持仙峰国际商业广尝华美立家建材家居广尝高桥商业城等商贸综合体项目加快建设，支持南丰县“西果东送”项目试点，推进“万村千乡市场工程”转型升级和商贸强镇示范工程。提升发展房地产业，促进房地产市场稳定健康发展，推进住宅小区物业管理市朝，发展建筑业总部经济，加快建筑企业“走出去”步伐，力争全市建筑业总产值增长20%以上，税收占地方财政收入比重达到10%以上。

培育发展新兴服务业。培育发展金融保险业，加大民间融资登记服务机构试点工作力度，力争引进1家大中型银行和1家村镇银行，组建3家以上县域农商银行，加大“财园信贷通”、“财政惠农信贷通”等金融支持实体经济力度，推动农业保险扩面提标，商业保险深度参与社保体系建设，支持江铃底盘海外上市，广德环保科技、川盛科技、江西麻姑实业“新三板”挂牌上市。培育发展电子商务，积极创建国家电子商务示范城市，支持各县(区)创建电子商务示范园区，加快发展农村电商，加大电商招商力度，支持电商重点项目建设，鼓励生产企业打造专业化电商平台，加大电商人才培养引进力度，支持职业院校人才培养基地建设，力争电子商务交易额达到200亿元。培育发展健康养老产业，积极开展全国养老服务业综合改革试点，大力发展居家养老服务，积极推行“医养结合”模式，推动健康养老与电商等信息化产业融合发展，推进县(区)福利院和农村敬老院升级改造项目建设。

(四)著力构建城乡统筹发展新格局。

坚持以人为本，顺应城镇化发展新趋势，著力打造宜居宜业的城乡环境。

提升城镇化发展水平。抓住我失籍制度改革实施意见出台的契机，积极放开市中心城区落户限制，有序推进农业转移人口市民化，加快推动新型城镇化健康、有序、可持续发展。围绕“融入南昌、对接海西、建设向莆经济带”，进一步优化城镇空间布局，做大做强市中心城区，增强要素和人口集聚功能。支持南城县依托区位优势和交通枢纽地位，建设副中心城市。鼓励以向莆线为主轴，以济广高速和抚吉高速为廊道，以市中心城区为核心，联动发展东乡、金溪、崇仁县城，积极培育城市组团，辐射带动宜黄、乐安、资溪、南丰、黎川、广昌等县城发展。

提升城乡基础设施建设水平。继续在全市实施百大重点工程，其中市中心城区基础设施项目86个，年内计划完成投资88亿元。尽快启动市中心城区东外环高速公路建设项目前期工作，积极推进市中心城区BRT项目。加快实施国省道升级改造工程，继续实施通村公路和危桥改造项目。开展主要建设工程抗震设防监督管理。加快推进40个中心镇、特色镇的建设，增强以镇带村的能力和水平。安排698个新农村建设村点，并确保60%以上为中心村。做好整乡整镇农网改造工作，全面启动农网改造升级工程前期工作。完成农田水利基本建设投资10.2亿元。坚持依法依规用地，积极推进城乡建设集约节约用地。多渠道筹措城市建设资金，力争市城投(集团)公司完成融资到位资金12亿元，用好土地增减挂政策，著力破解镇村联动建设资金难题。

提升城乡管理水平。继续推进“四城同创”，积极创建文明城市工作先进市。推进市中心城区生活垃圾焚烧发电项目，加快推进数字化城管系统工程建设、地下管线信息化工程和网上办案信息化管理。进一步完善联合执法机制，重点解决建筑工地扬尘污染、散装建材运输污染、车辆乱停乱放和非法营运等难点、热点问题，严厉打击“两违”。探索推进乡(镇)综合执法体制改革，在东乡县所辖全部乡镇、临川区所辖的上顿渡等6个乡(镇)先行试点，其他县(区)自主选择1―2个乡(镇)试点。

(五)切实打造生态文明建设新优势。

牢固树立“既要金山银山更要绿水青山、绿水青山就是金山银山”的理念，切实保护好、治理好、利用好生态，力争生态文明建设走在全省乃至全国的前列。

加强生态环境保护。严把建设项目环保准入关，做到“四个一律不批”，加强建设项目审批后监管，严把建设项目“三同时”验收关。将抚河、宜黄河作为重点监管区域，严控污染企业落户。积极实施封山育林和低产低效林改造，加大松材线虫和柑桔黄龙病防治力度。加强林地保护和自然保护区、生态功能保护区建设，抓好野生动植物和湿地保护工作，力争每个县(区)新增1个省级以上自然保护区、湿地公园或森林公园。推进抚河源国家生态补偿试点，积极争取武夷山脉核心县纳入国家重点生态功能区，支持东乡县建设APEC低碳示范城镇及海峡两岸低碳示范区。

加强生态环境治理。继续开展整治违法排污企业保障群众健康环保专项行动，对污染严重的企业实行挂牌督办，限期整改，依法查处未批先建、久试不验等违法行为。强化重金属污染防治，加强危险固废和危化品监管。强力推进主要污染物总量减排，确保完成省下达的污染减排计划任务。加快现有污水处理厂配套管网建设，尤其是老城区清污分流工程建设，提高污水收集率。加快推进工业园区和产业聚集区污水处理设施建设 金庸小说。探索有效的农村生活污水处理技术，完善农村生活垃圾清收集运模式，深入推进水库水质污染和禽畜养殖污染专项整治行动。

加快发展生态经济。加快发展生物医药、先进装备制造、电子信息、新能源等战略性新兴产业，大力发展环保节能等产业。依托良好的自然生态、丰富的山林水体资源，大力发展无公害农产品、绿色食品和有机农产品，油茶、毛竹、花卉、苗木和林下经济等林业产业，以及观光农业和旅游业，变生态优势为经济优势。(六)积极回应群众美好生活新期盼。

在稳增长、调结构、促改革的同时，以更大的力度惠民生，为全面建成小康社会夯实基矗

更加注重保障基本民生。继续稳定和扩大就业，推进全民创业。不断完善覆盖城乡的养老保障体系，进一步扩大社会保险覆盖面，落实好城镇基本医疗保险三条保障线，全面推行新农合大病保险和意外伤害保险。全面推进医药卫生体制改革，进一步加大卫生投入，推进市儿童专科医院新建、市第一人民医院老院区改扩建、市妇幼保健院扩建等项目，大力实施“卫生引智”工程。大力实施保障性住房安居工程，建设公租房4597套，完成各类棚户区和危旧房改造6339套，加大农村困难群众危房改造力度。逐步提高困难群众基本生活救济水平，进一步完善社会救助管理制度，推进申请低保家庭经济状况核对工作，加强城乡低保动态管理。大力实施精准扶贫，深入推进扶贫攻坚“六大工程”。加强食品检验检测体系建设，整合生产、流通环节食品监管职能，推进“放心粮油”六进工程，切实保障食品药品安全。加强集中式饮用水源地监管，实施好农村饮水安全工程，启动PM2.5监测并发布数据。

更加重视社会事业协调发展。围绕办人民满意教育目标，深化教育领域综合改革，推进教育“两区”建设，增加教育投入，扩大教育供给，做好义务教育就近入学工作，推进临川教育集团学校转型升级、职业教育改革发展，统筹推进各级各类教育均衡协调发展。大力弘扬临川文化，繁荣发展文化产业，推进基本公共文化服务标准化、均等化，加快博物馆、图书馆、文化馆、档案馆等公共文化设施数字化建设，加强各类文化遗产的保护、开发和利用，做好汤显祖逝世400周年纪念活动筹备工作。推进数字影院和广播电视“户户通”工程建设，实施全民健身计划，加强人口和计划生育工作，重视人才队伍建设。支持工商联、工会、共青团、妇联、科协、红十字会、残联、侨联、文联、社科联等人民团体及各民主党派、无党派、新阶层工作。

(七)全面落实依法治市新要求。

全面贯彻落实党的十八届四中全会精神，强化用法治的思维、手段和方式，调节经济运行，管理社会事务。

依法行使行政权力。严格在法律、法规和规章规定的权限范围内行使行政权力，坚持法定职责必须为、法无授权不可为。健全完善依法决策机制，完善决策风险评估机制，强化重大决策合法性审查，健全完善法律顾问制度。认真执行人大及其常委会决议决定，自觉接受监督，主动向人大及其常委会报告工作、向政协通报情况，认真办理人大代表建议和政协提案，广泛听取民主党派、工商联、无党派人士和人民团体意见。全面实行政务公开。

依法化解社会矛盾。强化法律在维护群众权益、化解社会矛盾中的权威，引导和支持群众理性合法表达诉求、依法信访、依法维护权益。畅通群众利益协调、权益保障的法治渠道，构建多元化社会矛盾化解机制。依法处理“以闹取利”的违法行为，坚决维护法治权威和尊严，切实纠正和防止“花钱买平安”的错误做法。推动领导干部带头学法守法用法，加强全社会普法教育。

依法推进社会治安综合治理。依法严厉打击危害人民群众生命财产安全的违法犯罪活动，坚决遏制严重刑事犯罪高发态势。重点整治社会治安混乱的地区、场所，改善治安状况，提升公众安全感。依法加强安全生产管理，保持安全生产形势总体平稳。开展公共安全隐患整治，严防发生危害公共安全的事件。创新社会治安防控体系，健全群防群治队伍，推进视频监控工程、社会治理信息平台建设。加强社区矫正工作，减少社会不和谐因素。

(八)努力开创政府自身建设新局面。

积极适应新常态，不断提高政府工作的能力和水平，切实把从严治党要求落实到从严治政中，为发展升级提供有力保障。

力践群众路线。巩固和拓展群众路线教育实践活动成果，将政府工作根植於群众的土壤之中。坚持密切联系群众，及时了解群众所思所想所盼，积极探索“群众点菜、政府买单”民生实事推进机制。坚持发展依靠群众，充分尊重群众的主体地位和首创精神，积极发挥人民群众的积极性和创造力。坚持发展为了群众，把更多精力用到关心群众生产生活中，把更多财力投入到解决群众最关心、最直接、最现实的利益问题上，使人民群众得到更多实惠。

力行廉洁从政。切实加强党风廉政建设，认真履行党风廉政建设责任制，落实好“一岗双责”。严格执行中央“八项规定”，厉行勤节约，严控一般性支出，确保“三公”经费支减不增。规范行政权力运行，坚决消除和防范权力寻租空间。建立公共资金、国有资产、国有资源和领导干部履行经济责任情况审计全覆盖制度，预防和治理腐败。构建多渠道、全方位的监督体系，以零容忍的态度严惩腐败。强化反腐倡廉教育和廉政文化建设，促使广大政府工作人员筑牢拒腐防变的思想道德防线，做到干部清正、政府清廉、政治清明。

力戒“为官不为”。加强宗旨意识和从政道德教育，引导干部在其位、谋其政、司其职、建其功。对“为官不为”问题开展明查暗访，围绕中心工作开展专项督查和效能监察。深入开展以治庸治懒治散为主要内容的专项整治活动，对“为官不为”问题进行认真核查、严肃问责。积极探索机关绩效管理与公务员薪酬制度改革挂钩，形成“干多干少不一样、干好干坏不一样”的激励效应。为想干事、能干事、干实事的干部营造良好环境。

各位代表，新的一年我们又站在了新的起点，迎来了新的机遇和挑战。让我们更加紧密地团结在以习近平同志为总书记的党中央周围，在省委、省政府和市委的坚强领导下，在市人大、市政协及社会各界的监督支持下，积极适应新常态，不断展示新作为，努力创造新业绩，开拓发展升级的新境界，为建设殷实、文明、和谐的幸福抚州而不懈奋斗!
